# Baseline Magnetic Resonance Imaging Assessment of Circumferential Resection Margin Predicts Long-term Survival in Rectal Adenocarcinoma: Experience from a Tertiary Care Center

**DOI:** 10.1007/s13193-025-02260-5

**Published:** 2025-04-25

**Authors:** Ambarish Chatterjee, Mufaddal Kazi, Mihir Chandarana, Ramkishan Nag, Suman Kumar Ankathi, Akshay Baheti, Vivek Sukumar, Ashwin Desouza, Avanish Saklani

**Affiliations:** 1 Unit of Colorectal Surgery, Department of Surgical Oncology, Tata Memorial Hospital, Homi Bhabha National Institute, Dr E Borges Road, Parel, Mumbai, 400012 India; 2 Department of Radiodiagnosis, Tata Memorial Hospital, Homi Bhabha National Institute, Dr E Borges Road, Parel, Mumbai, 400012 India; 3 Department of Surgical Oncology, Apollo Hospital, Nashik, 422003 India; 4 East Midlands, UK; 5 Specialty Surgical Oncology Hospital and Research Centre, Mumbai, 400086 India

**Keywords:** MRI in rectal cancer, Mesorectal fascia, Circumferential resection margin, Survival in rectal cancer, Rectal cancer surgery

## Abstract

In rectal adenocarcinoma, the diagnostic accuracy of baseline MRI for predicting circumferential resection margin (CRM) is established. However, data regarding the role of baseline and post-neoadjuvant chemoradiotherapy (NACTRT) MRI-mesorectal fascia (MRI-MRF)-positive status in predicting long-term oncological outcomes is relatively scarce and heterogeneous. The objective of the study is to evaluate the long-term oncological survival outcomes of baseline and post-neoadjuvant chemoradiation (NACTRT) MRI-MRF as predictors of long-term survival outcomes, i.e., overall survival (OS), disease-free survival (DFS), and locoregional recurrence-free survival (LRFS). Single center retrospective analysis from a prospectively maintained database. Patients undergoing curative surgery for rectal adenocarcinoma either upfront or post-NACTRT between July 2013 and April 2014. Patients with cT3/cT4 or N + received NACTRT before surgery. The pre-NACTRT MRI was recorded as MRI 1-MRF and post-NACTRT MRI was recorded as MRI 2-MRF. MRI scans done at presentation irrespective of further treatment were labeled as MRI T-MRF. Out of 254 patients, 217 were eligible for analysis. The median follow-up duration is 132 months. Seventy-six percent of patients received NACTRT. Overall, recurrences were seen in 68/217 (31.3%) patients, with 18 local and 50 distant recurrences. Eighty-six (39.6%) deaths were recorded, most due to disease progression. The 5-year OS of the cohort was 69.1% (95% C.I 63–75.8); 5-year DFS was 67.4% (95% C.I 61.2–74.3); and the 5-year LRFS was 91% (95% C.I 87–95.2). MRI T-MRF status was significantly associated in predicting OS, DFS, and LRFS. MRI 1-MRF status is a strong predictor for OS and DFS. The MRI 2-MRF status is a weak predictor for OS and is not associated with DFS and LRFS. The path-CRM-positive status is a significant predictor of OS and DFS, however not for LRFS. Baseline MRI-MRF status is a robust and strong predictor of long-term survival outcomes (OS, DFS, LRFS). Patients with baseline MRI-CRM-positive status have poorer outcomes irrespective of neoadjuvant therapy and poor histology features.

## Introduction

### Background

The survival and prognosis of rectal adenocarcinoma have traditionally been assessed by the AJCC-TNM staging system and histopathological parameters. In non-metastatic patients, pathological circumferential resection margin (path-CRM) strongly predicts local recurrence and survival [1, 2]. However, path-CRM and other strong prognostic factors, viz. pathological nodal status, and differentiation can only be obtained after resection. Accurate pre-operative assessment of circumferential resection margin (CRM), clinical nodal status is valuable in selecting patients not only for neoadjuvant therapy but also to prognosticate and provide a surgical roadmap. High-resolution magnetic resonance imaging (MRI) can delineate the local tumor extent and the relationship to the mesorectal fascia [3–6]. The diagnostic accuracy of pre-operative MRI for mesorectal fascia (MRI-MRF) involvement is well-established [7]. A positive mesorectal fascia (defined as tumor at MRF or ≤ 1 mm) is a known poor prognostic parameter for local recurrence, distant metastasis, and survival [1, 8, 9]. With the advent of modern MRI techniques, it is now possible to predict the CRM status on MRI (distance between the tumor and the mesorectal fascia) and select cases for neoadjuvant therapy and optimum surgery. With a good negative predictive value (NPV) and specificity, MRI remains a good tool to predict CRM status [10, 11]. However, the PPV of MRI to predict CRM is low, since the surgery, i.e., total mesorectal excision (TME) or extended-TME is tailored to achieve a negative path-CRM [12].

There is a scarcity of long-term data addressing MRI-MRF as a prognostic predictor in rectal adenocarcinoma patients [12–14]. The available literature consists of heterogeneous patients and lacks the details about whether the pre- or post- not neoadjuvant therapy MRI-MRF impact survival.

### Objectives

The objective of the study is to evaluate the long-term oncological survival outcomes of baseline- and post-neoadjuvant chemoradiation (NACTRT) MRI-MRF as predictors of long-term survival outcomes, i.e., overall survival (OS), disease-free survival (DFS), and locoregional recurrence-free survival (LRFS).

## Methods

### Study Design

The study is a retrospective analysis of a prospectively maintained database of rectal adenocarcinoma patients who underwent curative resections at our Institute. This study has received the institutional review board approval. The patients were exempted from providing informed consent because of the observational nature of the study, and participation did not imply any risk to the patients. This study followed the Strengthening the Reporting of Observational Studies in Epidemiology (STROBES) reporting guidelines.

### Setting

It is a single institute study conducted in a Tertiary Cancer Institute of India. Patients diagnosed with RA between 1st July 2013 and 30th April 2014 were recruited for the analysis. The follow-up data were obtained until December 2024. The details about demography, stage, tumor characteristics, neoadjuvant treatment, surgery, histopathology, adjuvant treatment, and follow-up were obtained systematically from the Hospital’s electronic medical record system and Centricity PACS (GE Healthcare, Milwaukee, USA) workstation.

At presentation, all patients underwent clinical examination, baseline blood investigations, tumor markers (CEA, CA-19.9), biopsy at the hospital or pathology review of biopsy performed elsewhere, a multi-detector contrast-enhanced computed tomography (CECT) scan of the thorax and abdomen, and MRI scan of the pelvis. A high-resolution MRI scan was obtained using 1.5 T (GE Healthcare, Milwaukee, USA), 1.5 T (Philips Medical Systems, Eindhoven, Netherlands), or 3 T (GE Healthcare, Milwaukee, USA) equipment using the institutional MRI rectum protocol. This protocol included large FOV (field of view) T1W and T2W axial and T2W sagittal sequences of the pelvis, small FOV thin oblique axial and oblique coronal T2W sequences along the plane of the rectum and axial diffusion-weighted images (DWI). In low rectal cancers, coronal and axial sections were obtained parallel and perpendicular to the anal canal. Rectal contrast or intravenous contrast was not administered in any of the patients.

After the initial evaluation, all patients underwent a multi-disciplinary tumor (MDT) board meeting which included a surgical-, radiation-, and medical-oncologist, and a radiologist dedicated to colorectal and gastrointestinal malignancies. All patients were assessed for either upfront surgery or post-NACTRT. Indications for NACTRT included T3/T4/cN + tumors or MRI-CRM + . The NACTRT protocol included 50 Gy in 25 fractions over 5 weeks along with concurrent oral capecitabine (850 mg/m^2^ twice daily from days 1–14 and 22–35). After 6–8 weeks of completion of NACTRT, a reassessment MRI was performed and further decision about surgery or chemotherapy was taken by MDT. All histopathological specimens were assessed for adequacy of total mesorectal excision (TME), CRM status, margins, LN status, NACTRT response/TRG, and differentiation.

MRI-MRF positive was defined as involvement of mesorectal fascia or tumor within 1 mm of MRF for upper- or mid-rectal tumors. For low-rectal cancers, a disease involving or within 1 mm of the inter-sphincteric plane or levator-ani muscle was considered as MRI-MRF positive. Lymph nodes (LNs) measuring ≥ 9 mm in short-axis were considered positive. Apart from size criteria, LNs were also assessed by shape, irregular borders, and heterogeneous signal intensity. LNs measuring 5–9 mm with any two criteria and LNs < 5 mm with all three criteria were considered positive [15]. A malignant LN within 1 mm or involving MRF or levator-ani was considered MRI-MRF positive.

For analysis, all MRI scans done at presentation irrespective of further treatment were labeled as MRI T. Patients undergoing NACTRT had two MRI scans: the pre-NACTRT scan was labeled as MRI 1 and the post-NACTRT scan as MRI 2. Pre-treatment MRI in all patients taken together MRI T = MRI (upfront surgery) + MRI 1 (pre-NACTRT). The MRI reports were reviewed for the study; the actual MRI images were not reviewed again.

After completion of treatment, at each follow-up, the patients underwent clinical evaluation and CEA and further imaging for suspicious findings. The follow-up protocol included visits every 3 months in the first 2 years, 6-monthly until 5 years, and annually thereafter.

### Participants

The inclusion criteria of this cohort study were patients with (i) tumor within 15 cm from the anal verge, (ii) curative-intent treatment, (iii) resectable cancer (upfront or after neoadjuvant therapy), and (iv) at least one MRI done as a part of workup. The exclusion criteria were (i) history of cancers at any site, (ii) locally advanced inoperable disease, (iii) metastatic disease, (iv) palliative intent treatment, and (v) MRI not done or details not available. During the study period, 254 patients were diagnosed with rectal adenocarcinoma, out of which 37 patients were excluded, and 217 patients were included in the analysis.

### Variables

As the study’s primary objective, the survival outcomes measured were OS, DFS, and LRFS. OS was calculated from the date of registration at the hospital to the death due to any cause or last follow-up. DFS was calculated from the date of registration to the date of disease recurrence (local/systemic) or death due to disease or last follow-up. LRFS was calculated from the date of registration to the date of local recurrence or last follow-up. Recurrence was defined as biopsy/FNAC-proven disease present locally or distant. Patients lost to follow-up were censored at the time of survival data accrual. Biopsy/FNAC was not done in case of obvious recurrence disease on the CECT scan. Local recurrence was defined as anastomotic site recurrence.

### Statistical Analysis

The patient demographic-, clinical-, treatment-, and surgical-related variables were analyzed and presented as frequencies (percentage), mean, or median as appropriate. The categorical variables were analyzed using the chi-square test or Fischer’s exact test with a *p*-value of less than 0.05 considered to be statistically significant. The survival analysis was done using the Kaplan–Meier method, and groups were compared using the log-rank test. The Cox regression method was used for univariate analysis. The statistical analysis was done with SPSS (IBM Corp. Released 2019. IBM SPSS Statistics for Windows, Version 26.0. Armonk, NY: IBM Corp.).

## Results

### Demographic and Clinical Outcomes

**Fig. 1:**
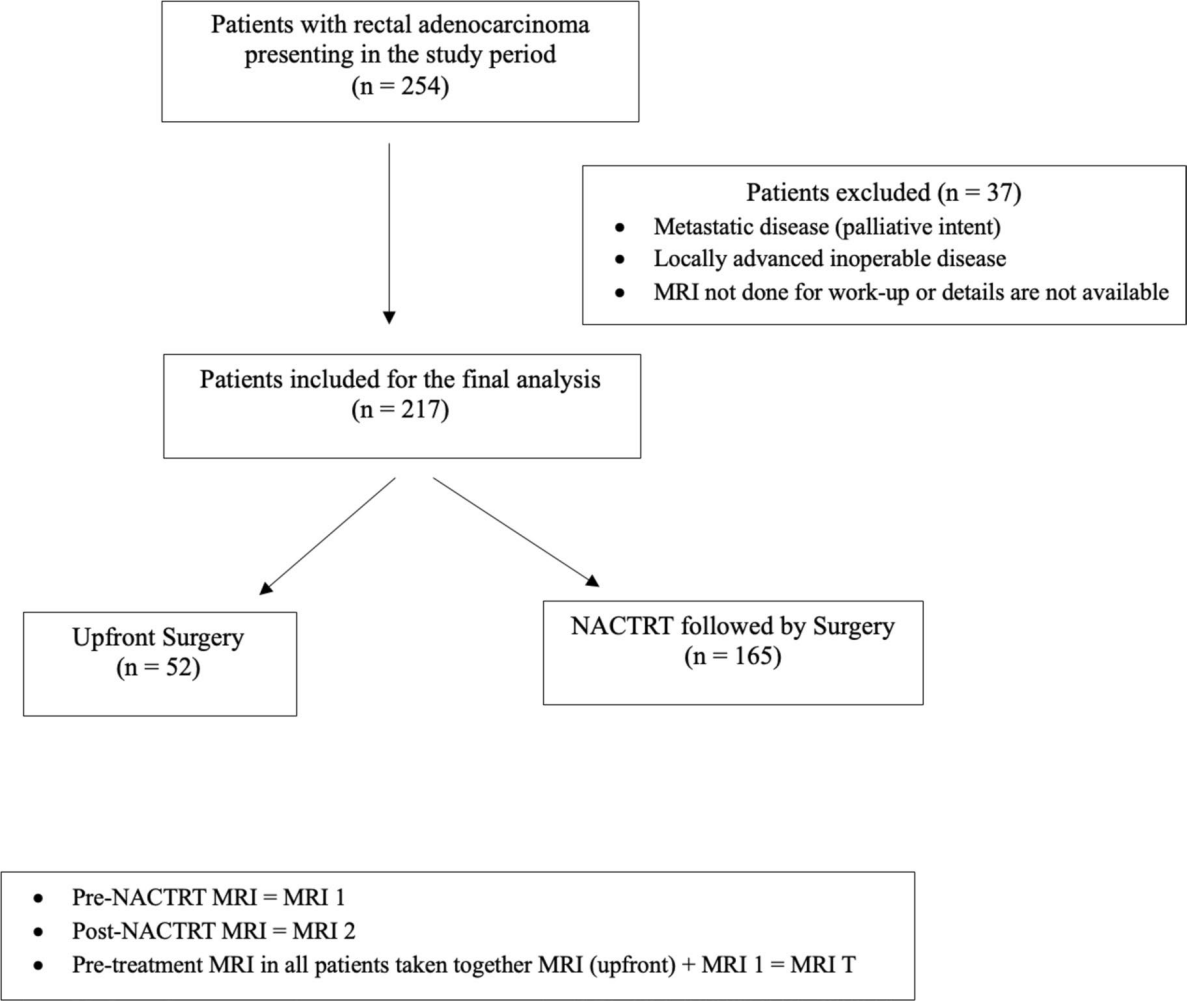
Consort diagram

The total number of patients diagnosed during the study period was 254, out of which 217 patients were eligible for the study (Fig. 1). The mean age of the cohort was 48 (36–58) years with predominantly males (2:1). Out of 217 patients, 165 (76%) received NACTRT before undergoing surgery. The rate of a negative CRM margin status of 95.9%. Fifty-two patients (24%) patients had aggressive histology (poorly differentiated, and signet-ring) (Table 1).

**Table 1:** Clinical characteristics

Characteristics	*n* = 217
Age	48 years (36–58)
Sex	
Female	72 (33%)
Male	145 (67%)
Histology	
Well-differentiated and moderately differentiated	165 (76%)
Poorly differentiated and signet-ring cell adenocarcinoma	52 (24%)
Pre-op T stage	
T1	4 (1.8%)
T2	46 (21%)
T3	152 (70%)
T4	15 (6.9%)
NACTRT	165 (76%)
MRI T-MRF positive	73 (34%)
MRI 1 positive	
No	96 (44%)
Yes	69 (32%)
NA (upfront)	52 (24%)
MRI 2-MRF positive	
No	101 (47%)
Yes	64 (29%)
NA (upfront)	52 (24%)
Path-CRM positive	9 (4.1%)
Local recurrence	18 (8.3%)
Any recurrence	68 (31.3%)
Death	86 (39.6%)
Follow-up (median)	132 months
5-year OS	69.1% (63–75.8)
5-year DFS	67.4% (61.2–74.3)
5-year LRFS	91% (87–95.2)

*NACTRT*, neoadjuvant chemoradiation; *MRF*, mesorectal fascia; *NA*, not applicable; *CRM*, circumferential resection margin; *OS*, overall survival; *DFS*, disease-free survival; *LRFS*, local recurrence-free survival

### Survival Outcomes

The median follow-up duration of the study was 132 months. Overall, recurrences were seen in 68/217 (31.3%) patients, with 18 local and 50 distant recurrences. Eight-six (39.6%) deaths were recorded, most due to disease progression. The 5-year OS of the cohort was 69.1% (95% C.I 63–75.8); 5-year DFS was 67.4% (95% C.I 61.2–74.3); and the 5-year LRFS was 91% (95% C.I 87–95.2) (Table 1).

### Impact of MRI-CRMs on Survival Outcomes

#### Overall Survival (OS)

**Fig. 2:**
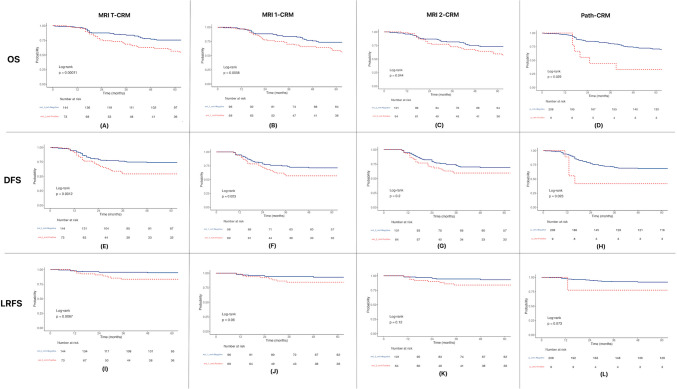
Survival graphs

The MRI T-MRF positivity had a statistically significant association with OS (*p* = 0.00011) (Fig. 2A). In patients who underwent NACTRT, MRI 1-MRF positivity had a statistically significant association with OS (*p* = 0.0058) (Fig. 2B). Post-NACTRT MRI 2-MRF positivity had a statistically significant association with OS (*p* = 0.044) (Fig. 2C). The path-CRM-positive status also had a statistically significant association with OS (*p* = 0.029) (Fig. 2D).

#### Disease-Free Survival (DFS)

The MRI T-MRF and MRI 1-MRF positivity had a statistically significant association with DFS; *p* = 0.0012 and *p* = 0.023 respectively (Fig. 2E, 2F). The MRI 2-MRF positivity did not have a statistically significant association with DFS; *p* = 0.2 (Fig. 2G). The path-CRM-positive status did have a statistically significant association with DFS; *p* = 0.023 (Fig. 2H).

#### Locoregional Recurrence-Free Survival (LRFS)

The MRI T-MRF-positive status had a statistically significant association with LRFS; *p* = 0.0067 (Fig. 2I). The MRI 1-MRF (*p* = 0.06)- and MRI 2-MRF (*p* = 0.12)- and path-CRM (*p* = 0.073)-positive status did not have a statistically significant association with LRFS (Fig. 2J, 2K, 2L).

### Univariate Analysis

The univariate analysis using Cox regression analysis is depicted in Table 2. In univariate analysis, MRI T-MRF-positive status is independently associated with OS, DFS, and LRFS. The MRI 1-MRF positivity was significantly associated with OS and DFS, and not LRFS. The MRI 2-MRF-positive status was significantly associated with OS alone. The path-CRM positivity was significantly associated with OS and DFS, but not LRFS.

**Table 2:** Univariate analysis

	**Overall survival HR**	**Disease-free survival HR**	**Local recurrence-free survival HR**
MRI T-MRF positive	2.25 (1.47–3.44, *p* < 0.001)	2.15 (1.33–3.46, *p* = 0.002)	3.43 (1.33–8.86, *p* = 0.011)
MRI 1-MRF positive	1.88 (1.18–2.99, *p* = 0.008)	1.80 (1.06–3.04, *p* = 0.029)	2.52 (0.92–6.95, *p* = 0.073)
MRI 2-MRF positive	1.59 (1.00–2.53, *p* = 0.048)	1.40 (0.83–2.37, *p* = 0.211)	2.14 (0.80–5.74, *p* = 0.132)
Path-CRM positive	2.47 (1.08–5.68, *p* = 0.033)	2.82 (1.13–7.04, *p* = 0.026)	3.58 (0.82–15.68, *p* = 0.090)

*HR*, hazard ratio

## Discussion

### Key Results

The objective of the study was to evaluate the long-term oncological survival outcomes of baseline- and post-neoadjuvant chemoradiation (NACTRT) MRI-MRF-positive status as predictors of long-term survival outcomes.

Through the study, using Kaplan-Meir method and Cox regression analysis, we found that baseline MRI-MRF status (positive vs negative) is significantly associated in predicting OS, DFS, and LRFS. In patients receiving neoadjuvant chemoradiation, the pre-NACTRT MRI-MRF status (positive vs negative) is a strong predictor for OS and DFS but not LRFS. The post-NACTRT MRI-MRF status (positive vs negative) is a weak predictor for OS, although statistically significant, and is not associated with DFS and LRFS. The path-CRM-positive status is a significant predictor of OS and DFS, however not for LRFS.

It could reflect that those patients with a positive MRF status on baseline (MRI T or MRI 1) are aggressive cancers, to begin with, and carry poorer prognosis. Since approximately 30% of all rectal cancers are locally advanced at presentation, identifying these subsets of patients with positive MRI-MRF status at the baseline (MRI T or MRI 1) is important to offer them early and intensive neoadjuvant therapy, and prognostic them. Also, an attempt to identify patients early in the course of disease through screening programs looks to be a promising approach.

Although MRI 2-MRF status was a weak predictor of OS and not significantly associated with DFS and LRFS, its importance cannot be understated. Post-NACTRT MRI (MRI 2-MRF) status identifies patients who have responded to NACTRT and are operable with standard TME to achieve a negative path-CRM. It also identifies patients who have involved MRF (post-NACTRT) and need further neoadjuvant therapy or extended TME to achieve wider margins and a negative path-CRM. Post-treatment fibrosis in the MRF reduces the accuracy of MRI to predict MRF-positive status. The fact that because surgical resections are planned based on MRI 2-MRF status (positive vs negative), it may not be correlating with DFS and LRFS. Also, since neoadjuvant therapy improves local recurrence rates and survival [16, 17], the post-therapy MRI 2-MRF status will not impact DFS and LRFS. MRI 2-MRF also identifies patients who have completed NACTRT and have a complete clinical-radiological response and can thus be kept in wait and watch protocol. Hence, once the decision is made to give NACTRT, post-NACTRT MRI (MRI 2) is strongly recommended [18].

The secondary analysis of the OPRA trial demonstrated that baseline MRF involvement independently predicted likelihood of organ preservation (HR = 1.45, 95% C.I = 1.01–2.09) and DFS (HR = 2.02, 95% C.I = 1.26–3.26) [19]. A study conducted by Chin et al. evaluating organ preservation after short course radiation and consolidation chemotherapy concluded that CRM involvement in MRI is a strong predictor of non-complete response [20]. These studies bolster the importance of baseline MRI as a prognostic predictor in organ preservation, and also signify the importance of post-treatment MRI.

In the study conducted by Taylor et al. from the MERCURY group, both MRI-MRF involvement and path-CRM were good predictors for OS, DFS, and local recurrence. However, the authors did not analyze the pre-NACTRT MRI and post-NACTRT MRI separately. Also, in that study, only 29% received NACTRT [13]. In our study, majority of the patients received NACTRT (76%) and the remaining patients did not receive NACTRT because based on clinical-radiological parameters, they were ineligible for NACTRT. This suggests that poorer survival outcomes and distant failure rates are seen in MRI-MRF-positive patients even in patients receiving NACTRT which in itself is a strong prognostic factor. The importance of path-CRM as a prognostic tool is known. In a recent analysis by Patel et al. evaluating the outcomes of path-CRM-positive patients, the authors found that 69.7% of patients have distant recurrences and out of these, only 20.75% of patients could be salvaged [21]. In our study, path-CRM-positive status had an impact on OS and DFS.

### Strengths and Limitations

This is a large single-center robust data with the long-term follow-up that shows the value of baseline MRI in predicting the overall survival in rectal adenocarcinoma patients. The cohort of patients was homogeneous and the post-NACTRT MRI-MRF was also analyzed in the same set of patients making it more homogeneous and robust. The proportion of patients with aggressive histology was 24% which is substantial and it would be intriguing to know the MRI predicted outcomes of this subset of patients. The study is not without limitations. First, although this is a prospectively maintained database, the study is a retrospective and has an inherent selection bias. Second, other factors like distance from the anal verge, extramural vascular invasion, tumor regression grade, and sphincter involvement are not available for analysis which influences survival and decision-making. Third, we did not include patients who received short-course radiotherapy with or without neoadjuvant chemotherapy, and the impact of adjuvant chemotherapy in these patients.

## Conclusion

In this study, we demonstrated that the baseline MRI-MRF status is a very strong predictor of long-term survival in rectal adenocarcinoma patients. The patients with baseline MRI-MRF-positive status have particularly poorer outcomes irrespective of neoadjuvant therapy and poor histology features. These patients need neoadjuvant therapy, surgery, and adjuvant treatment tailored based on the MRI, proximity and infiltration of adjacent structures and a close and stringent follow-up. The MRI-MRF status can be incorporated in the AJCC staging system for better prognostication. Furthermore, multi-centric studies with larger patient numbers and data will be more useful.
